# Supplementary material for: The Reliability and Compatibility of the Paper and Electronic Versions of the POLLEK Cohort Study Questionnaire
Source: Healthcare (Basel). 2020 Oct 29;8(4):438. doi: 10.3390/healthcare8040438 (PMC7711888; doi:10.3390/healthcare8040438)
Supplement: Supplementary file 1 [file healthcare-08-00438-s001.pdf]

healthcare

MDPI

Table S1. Repeatability of answers to particular questions.

Selected** General Questions	κ	95% CI	ϱ	τ
M1. Gender	0.96	0.93-0.99	0.96	0.96
M2. Age [years]	0.98	0.96-1	1	0.99
M3. Marital status	0.96	0.93-0.99	0.96	0.96
M8A. Source of income-dependent on parents	0.76	0.58-0.95	0.78	0.78
M8B. Source of income-scholarship	0.78	0.64-0.91	0.79	0.79
M8C. Source of income-pension or allowance	0.83	0.66-0.99	0.84	0.84
M8D. Source of income-earning work	0.83	0.74-0.93	0.84	0.84
M8E. Source of income-other	0.48	0.33-0.64	0.57	0.57
M9. Current financial situation	0.87	0.82-0.91	0.85	0.84
M9. Current financial situation [4 categories]	0.87	0.82-0.91	0.85	0.84
M9. Current financial situation [3 categories]	0.87	0.83-0.92	0.87	0.86
M10. Current place of residence during studies	0.97	0.95-1	0.98	0.98
M11. Have you ever smoked so-called traditional cigarettes?	0.92	0.87-0.96	0.92	0.92
M12. Have you ever smoked electronic cigarettes?	0.95	0.91-0.98	0.95	0.95
M14. Do you take up sports activities to improve physical fitness?	0.97	0.95-0.99	0.98	0.98
M15. How do you assess the current state of your health?	0.77	0.71-0.82	0.86	0.83
M16. Have you ever been diagnosed with a chronic disease by a doctor?	0.95	0.9-0.99	0.95	0.95
M16_2. If yes, are you under constant medical care?	0.96	0.9-1	0.97	0.97
**Nutrition**	**κ**	**95% CI**	**ϱ**	**τ**
M17. How many meals do you eat per day?	0.91	0.87-0.95	0.94	0.93
M18. How many meals do you eat that contain animal protein?	0.87	0.82-0.92	0.89	0.87
M19. How often do you consume milk or milk products?	0.88	0.84-0.93	0.89	0.88
M20. How often do you eat fruit and vegetables?	0.89	0.85-0.93	0.92	0.91
M21. How often do you eat vegetable or fruit salads?	0.86	0.81-0.91	0.91	0.89
**WHOQOL-BREF**	**κ**	**95% CI**	**ϱ**	**τ**
B1. How would you rate your quality of life	0.78	0.73-0.84	0.86	0.84
B2. How satisfied are you with your health	0.82	0.77-0.87	0.89	0.86
B3. To what extent do you feel that physical pain prevents you from doing what you need to do	0.84	0.79-0.89	0.88	0.86
B4. How much do you need any medical treatment to function in your daily life	0.79	0.73-0.85	0.87	0.84
B5. How much do you enjoy life	0.81	0.76-0.86	0.87	0.85
B6. To what extent do you feel your life to be meaningful	0.86	0.82-0.9	0.92	0.9

B7. How well are you able to concentrate	0.81	0.76-0.86	0.89	0.87
B8. How safe do you feel in your daily life	0.8	0.74-0.85	0.87	0.84
B9. How healthy is your physical environment	0.81	0.76-0.86	0.87	0.84
B10. Do you have enough energy for everyday life	0.76	0.7-0.81	0.85	0.82
B11. Are you able to accept your bodily appearance	0.9	0.87-0.94	0.93	0.92
B12. Have you enough money to meet your needs	0.82	0.77-0.87	0.89	0.87
B13. How available to you is the information that you need in your day-to-day life	0.78	0.72-0.83	0.85	0.83
B14. To what extent do you have the opportunity for leisure activities	0.79	0.74-0.84	0.92	0.88
B15. How well are you able to get around	0.7	0.64-0.76	0.84	0.8
B16. How satisfied are you with your sleep	0.86	0.82-0.91	0.94	0.92
B17. How satisfied are you with your ability to perform your daily living activities	0.77	0.72-0.82	0.85	0.82
B18. How satisfied are you with your capacity for work	0.76	0.7-0.81	0.88	0.85
B19. How satisfied are you with yourself	0.78	0.73-0.83	0.86	0.83
B20. How satisfied are you with your personal relationships	0.77	0.72-0.83	0.86	0.83
B21. How satisfied are you with your sex life	0.89	0.85-0.93	0.96	0.94
B22. How satisfied are you with the support you get from your friends	0.82	0.77-0.87	0.9	0.88
B23. How satisfied are you with the conditions of your living place	0.85	0.81-0.9	0.92	0.9
B24. How satisfied are you with your access to health services	0.79	0.74-0.85	0.88	0.85
B25. How satisfied are you with your transport	0.87	0.83-0.91	0.91	0.9
B26. How often do you have negative feelings such as blue mood, despair, anxiety, depression	0.8	0.75-0.85	0.83	0.81
**Alcohol Use Disorder Identification Test (AUDIT)**	**κ**	**95% CI**	**ϱ***	**τ***
A1. How often do you have a drink containing alcohol?	0.86	0.82-0.91	0.92	0.9
A2. How many drinks containing alcohol do you have on a typical day when you are drinking?	0.9	0.86-0.93	0.95	0.94
A3. How often do you have six or more drinks on one occasion?	0.84	0.79-0.89	0.91	0.89
A4. How often during the last year have you found that you were not able to stop drinking once you had started?	0.77	0.69-0.86	0.87	0.86
A5. How often during the last year have you failed to do what was normally expected from you because of drinking?	0.84	0.78-0.89	0.87	0.86
A6. How often during the last year have you needed a first drink in the morning to get yourself going after a heavy drinking session?	0.81	0.71-0.9	0.83	0.82
A7. How often during the last year have you had a feeling of guilt or remorse after drinking?	0.79	0.73-0.85	0.83	0.82
A8. How often during the last year have you been unable to remember what happened the night before because you had been drinking?	0.9	0.85-0.95	0.92	0.91
A9. Have you or someone else been injured as a result of your drinking?	0.97	0.93-1	0.98	0.97
A10. Has a relative or friend or a doctor or another health worker been concerned about your drinking or suggested you cut down?	0.81	0.69-0.92	0.83	0.83

Legend: κ—unweighted Cohen's *kappa*; CI—confidence interval; ϱ—Spearman's *rho*; τ—Kendall's *tau; *—all* τ and ϱ significant at *p <0.0001;* **—appropriate for analysis.
